# Right middle cerebral artery aneurysm posted for clipping on dual anti-platelet therapy

**DOI:** 10.4103/0019-5049.60510

**Published:** 2010

**Authors:** Satyen Parida, Sandeep Kumar Mishra, K Sudeeep, Aahok Shankar Badhe

**Affiliations:** Department of Anaesthesiology and Critical Care, Jawaharlal Institute of Post-Graduate Medical Education and Research, Pondicherry, India

Sir,

Current recommendations regarding patients on dual antiplatelet therapy[1,2] (Aspirin and Clopidogrel) presenting for surgery within a closed space (e.g., intracranial, spinal canal, posterior chamber of eye) are to stop Clopidogrel for at least 5 to 7 days prior to proposed surgery. Aspirin, if it is part of a dual antiplatelet regime, can be continued.

Cerebral aneurysms manifesting as subarachnoid haemorrhage (SAH) are associated with significant morbidity (50% amongst survivors) and mortality (25%). Rebleeds are common complications after aneurysmal SAH. The risk of rebleed is maximal, viz., at 4%, within the first 24 hours after SAH. After 48 hours, the risk increases by 1.5% everyday. At the end of 2 weeks, the total risk is around 19%.[3] Owing to risks of rebleeding, aneurysmal clipping within 0-3 days is strongly recommended for patients with well-preserved cognitive functions. The results of the International Cooperative Study on the Timing of Aneurysm Surgery (ICSTAS)[4,5] suggested identical mortality of around 20% and favourable outcomes of about 60% for surgeries conducted either between 0 and 3 days or 11 and 14 days post-SAH. The worst outcomes and maximal mortality rates were for surgeries conducted between 7 and 10 days post-SAH. Patients with preserved neurological function did best when operated early.

A 68-year-old lady presented with severe headache of 3 days' duration, chest pain and altered sensorium. ECG features of anterolateral ischaemia had led to her being started on Aspirin and Clopidogrel. Glasgow Coma Scale (GCS) was 13/15. CT brain revealed right temporal haematoma with sulcal SAH. CT angio showed a right middle cerebral artery (MCA) aneurysm at M1-M2 junction. Antiplatelet medications were stopped, pharmacological anti-cerebral oedema measures instituted, and the patient was worked up for aneurysmal clipping. She was hypertensive for previous 3 years, on Amlodipine and Atenolol. Amlodipine was replaced with Nimodipine. Haemato-biochemical investigations were unremarkable except for International normalized ratio (INR) of 3.3. Surgery was postponed and patient transfused 4 units of Frozen Plasma (FFPs) and 4 units of platelet concentrates. Repeat INR was 1.3. Patient's GCS had improved to 15/15. She was posted for clipping of aneurysm on the sixth day of the SAH, which was 4 days after omission of aspirin and Clopidogrel. Four units of platelets were kept ready for use if necessary perioperatively. However, surgery was uneventful and concluded with a sub-galeal suction drain. The patient recovered fully and was extubated on table. Postoperative course of the patient was unremarkable.

Most guidelines agree that Clopidogrel be stopped at least 5 to 7 days prior to elective surgery if possible. However, for a situation such as the one we found ourselves in, wherein the risks of rebleed, vasospasm and suboptimal outcomes increase with each passing day of waiting, the approach is far from clear. With regard to Clopidogrel, pharmacological evidence does suggest a return of platelet function from day 3 after cessation of the drug, possibly due to formation of new platelets.[6] Hence we decided to take the patient up for surgery on the fourth day of omission of antiplatelet agents, after platelet transfusions and optimisation of other bleeding parameters. Clearly, a balance needs to be addressed between potential risks of perioperative bleeding and the risks associated with delaying surgery, especially in semi-emergency situations, even in neurosurgical procedures when Patient is on clopidogrel.
